# Stress Test-Induced Left Bundle Branch Block

**DOI:** 10.7759/cureus.17384

**Published:** 2021-08-23

**Authors:** Mackenzie D Hamilton, Ebubechukwu Ezeh, Mohamed Suliman, John Saylor, Ellen Thompson

**Affiliations:** 1 Internal Medicine, Marshall University Joan C. Edwards School of Medicine, Huntington, USA; 2 Cardiology, Marshall University Joan C. Edwards School of Medicine, Huntington, USA

**Keywords:** lbbb, stress test, cardiology, mpi, regadenoson

## Abstract

Left bundle branch block (LBBB) is an uncommon complication of myocardial perfusion imaging using Regadenoson, a vasodilatory agent. The mechanism is poorly understood at this time but could be related to ongoing ischemia. The use of a Regadenoson with the subsequent occurrence of LBBB could be a predictor of coronary artery disease or conduction abnormalities and should be understood by the physician to diagnose and risk stratify patients undergoing myocardial perfusion imaging properly.

## Introduction

Myocardial perfusion imaging (MPI) stress test is performed using exercise or vasodilatory agents such as dipyridamole (a vasodilatory agent which achieves vasodilation through the activity of adenosine deaminase and phosphodiesterase) or Regadenoson (Increases coronary blood flow through an adenosine receptor agonist, thus, mimicking exercise). Regadenoson is also known to cause significantly higher peak, absolute and relative heart rates than dipyridamole during MPI. The incidence of exercise stress test-induced left bundle branch block (LBBB) is rare and reportedly occurs in approximately 0.5-1% of all patients undergoing exercise testing [1]. The mechanism is poorly understood, but ischemia is one of the proposed etiologies. We present a case of a 56-year-old Caucasian male who had a transient LBBB during stress MPI using Regadenoson.

## Case presentation

**Figure 1:**
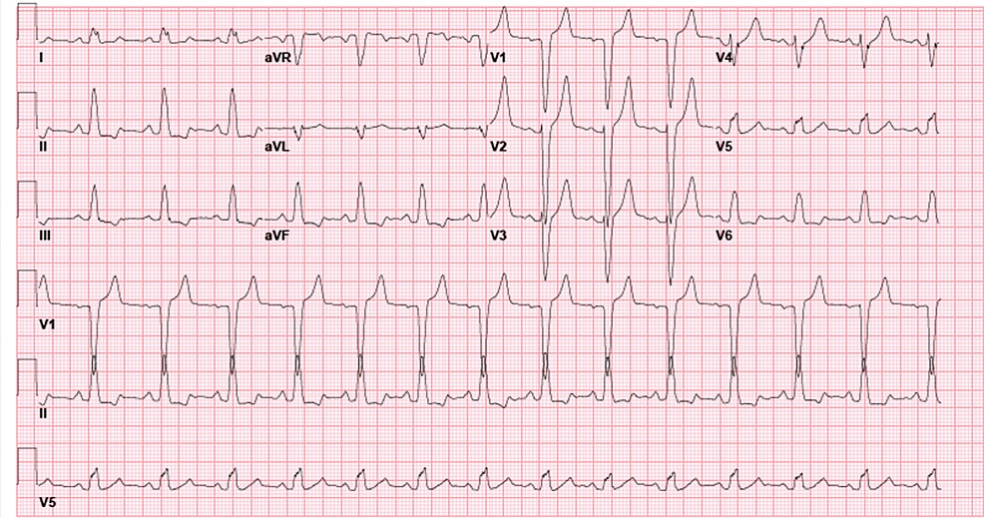
EKG with new LBBB LBBB: Left bundle branch block

**Figure 2:**
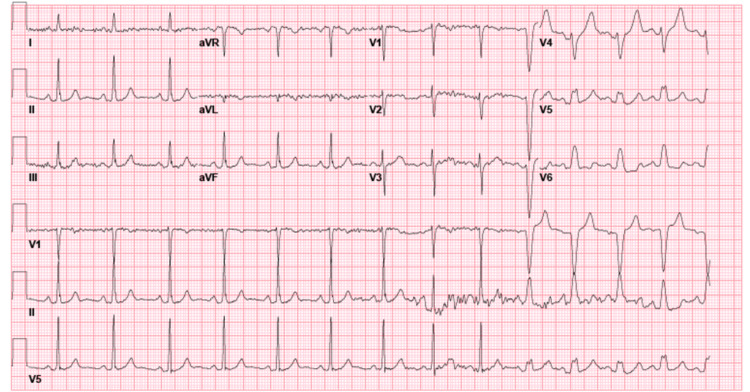
Electrocardiogram 18 days prior to presentation without LBBB LBBB: Left bundle branch block

**Figure 3:**
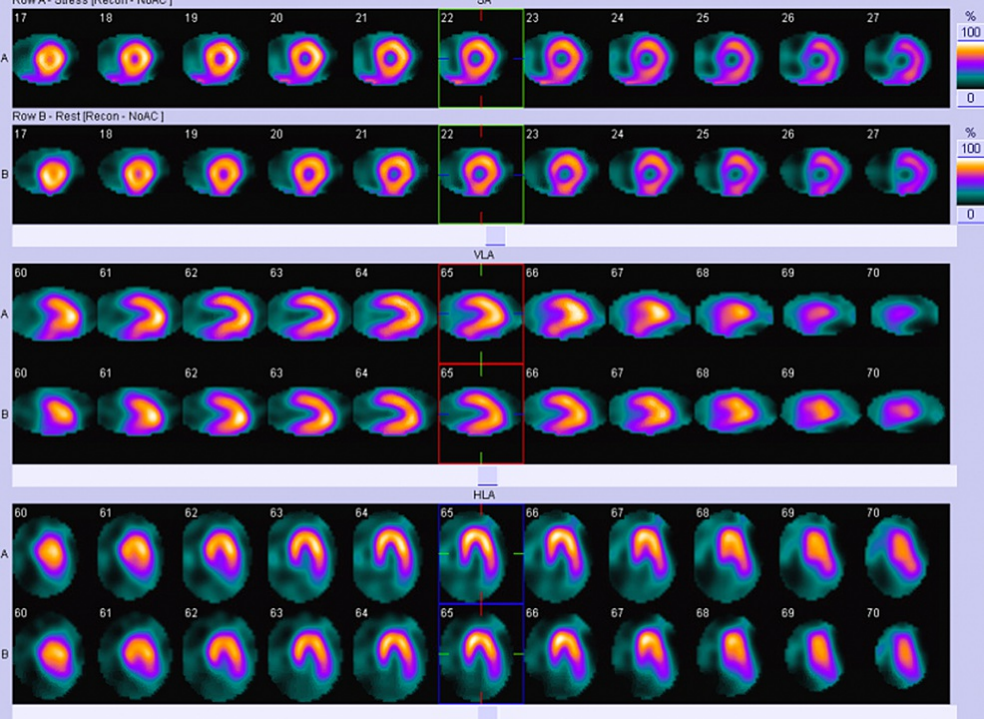
Technetium 99m Myoview

We present a 56-year-old Caucasian male with a past medical history of hypertension and hyperlipidemia presenting with a one-month history of atypical chest pain. The patient specified that the pain was a burning sensation along the right side of the chest without aggravating or alleviating factors. The patient admitted to numbness and tingling of his right hand in an ulnar nerve distribution and denied shortness of breath. His social history was remarkable for chewing tobacco for over 50 years and a strong family history of myocardial infarction, stating his mother had two heart attacks before the age of 45. With a baseline heart rate of 84, the patient subsequently underwent treadmill exercise stress testing and was found to have new-onset LBBB (Figure 1) at a heart rate of 129/min; during this time, the patient was asymptomatic. The most recent electrocardiogram (EKG) 18 days prior revealed sinus rhythm without sign of LBBB (Figure 2). Due to the EKG changes, the patient was admitted for further workup and ruled out acute coronary syndrome with a Regadenoson stress test. While in the hospital, an electrocardiogram was unremarkable for LBBB changes, troponin trend was unremarkable. The following day, Regadenoson stress testing was performed. The patient received 0.4mg of intravenous Regadenoson over 10-15 seconds, followed by a stress dose of Technetium 99m for Myoview (Figure 3). The patient developed transient LBBB after injection of Regadenoson which resolved at the end of the stress test; he was asymptomatic throughout the procedure. The patient was transferred to a percutaneous coronary intervention capable facility; left heart catheterization was unremarkable for coronary disease.

## Discussion

The presence of LBBB on a resting electrocardiogram (ECG) and at low work levels on a stress test essentially exclude exercise-induced LBBB [2]. EI-LBBB is an uncommon phenomenon that compromises the interpretability of ECG. The exact mechanism for EI-LBBB remains unclear, but it may reflect underlying perfusion defect or structural heart disease [2]. When patients develop EI-LBBB, MPI with a vasodilatory agent is usually indicated. We, therefore, pursued MPI stress testing in our patient who initially developed EI-LBBB.

When LBBB occurs, it may produce a partially reversible or fixed septal defect on perfusion imaging [3]. This perfusion defect is believed to be induced by tachycardia [1]. The proposed mechanism is not fully understood, but it is believed to result from reduced diastolic blood flow caused by delayed septal contraction. Blood flow is further compromised by tachycardia since the duration of diastole is shortened [4]. For this reason, stress tests that do not promote tachycardia are preferably selected in patients with baseline LBBB. Similarly, Regadenoson increases HR and thus causes LBBB more so than adenosine and dipyridamole [5]. The mechanism for Regadenoson-induced tachycardia is likely due to sympathoexcitation since it increases serum norepinephrine and epinephrine levels even though it is a coronary vasodilator [2]. Our patient had developed transient tachycardia and LBBB upon administration of Regadenoson probably from the sympathetic effects of Regadenoson. This HR response seen after Regadenoson administration may be blunted in people with diabetes, likely from sympathetic denervation; this supports the sympathoexcitation hypothesis [1]. Our patient was not diabetic and thus had the complete HR response, which probably predisposed him to LBBB.

Some studies have documented a strong association between stress-induced LBBB and coronary artery disease (CAD) or heart failure [6]. Such patients may benefit from coronary angiography (CA). Our patient had CA performed, which came back unremarkable. In the same study, the individuals who developed EI-LBBB were older and had a higher prevalence of CAD, heart failure, diabetes, and tobacco use which may have impacted the findings in the study. The HR at which LBBB occurs may predict the presence of CAD. The onset of LBBB at HR of 125/min or lower has been reported to strongly correlate with the presence of occlusive coronary artery disease (CAD), compared to patients who develop LBBB at HR above 125/min who show normal coronary arteriograms and have a better prognosis [7]. The findings in our patient support this. Our patient developed LBBB at a heart rate above 125/min and subsequently underwent a coronary angiogram which showed non-occlusive disease. Furthermore, patients with EI‑LBBB are reported to be at increased risk of developing permanent LBBB, ventricular dyssynchrony, and dysfunction, all of which require close follow-up [6].

## Conclusions

LBBB is an uncommon complication of Regadenoson stress tests and should be recognized as it may predict the presence of CAD and the probability of permanent LBBB and conduction abnormalities. When this occurs, patients may benefit from CA to rule out existing CAD.
